# LncRNA TUG1 promotes hypertrophic scar formation via the miR-627/IGF1R axis

**DOI:** 10.1007/s10735-026-10814-2

**Published:** 2026-06-06

**Authors:** Zeming Bai, Ziyang Han, Xiangzi Kong, Rirui Zhao, Haiwei Sun, Hai Huang, GuYue Wang, Zhe Li, Kai Tao, Jiulong Liang

**Affiliations:** Burn and Plastic Surgery Department, General Hospital of Northern Theater Command, Shenyang, 110000 China

**Keywords:** Hypertrophic scar, LncRNA TUG1, miR-627, IGF1R, Fibrosis

## Abstract

Hypertrophic scars (HS) are fibrotic skin disorders driven by abnormal fibroblast activity. The molecular mechanisms underlying HS remain incompletely understood, particularly the role of non-coding RNAs. Expression levels of lncRNA TUG1, miR-627, and IGF1R were measured in HS tissues and fibroblasts using qRT-PCR and Western blotting. Dual-luciferase assays validated direct interactions. Functional effects of TUG1 and miR-627 on fibroblast proliferation and migration were assessed using MTT and Transwell assays. A rabbit ear model of HS was used to examine in vivo effects of TUG1 and miR-627 modulation on scar formation and molecular expression. TUG1 was significantly upregulated in HS tissues and inversely correlated with miR-627, which was downregulated. TUG1 promoted fibroblast proliferation and migration by directly sponging miR-627, thereby lifting repression on IGF1R, a known pro-fibrotic effector. Luciferase assays confirmed direct binding of miR-627 to both TUG1 and IGF1R. Co-transfection of miR-627 attenuated TUG1-induced IGF1R upregulation and reversed its pro-fibrotic cellular effects. In vivo, TUG1 overexpression led to increased scar thickness, collagen deposition, and IGF1R expression, while miR-627 overexpression mitigated these effects. Co-administration of both restored scar morphology and molecular markers to near-control levels. TUG1 promotes hypertrophic scar formation by sponging miR-627 and derepressing IGF1R. This newly identified TUG1–miR-627–IGF1R axis plays a central role in HS pathogenesis and may serve as a promising therapeutic target for fibrotic skin disease.

## Introduction

Hypertrophic scar (HS) represents a considerable clinical challenge, characterized by abnormal extracellular matrix deposition and excessive fibroblast proliferation (Oosterhoff et al. 2021, Barone et al. 2021). HS develops in up to 70% of patients following deep dermal burns and is frequently associated with persistent symptoms such as pain, erythema, pruritus, and contractures, which can severely impair physical function (Finnerty et al. 2016). Management of HS remains difficult due to its high recurrence rate, reported to exceed 50% after burn injuries, and the need for long-term, resource-intensive treatment (Nischwitz et al. 2020). Despite several treatment options such as surgery, radiation, and laser therapy, none have demonstrated consistently effective outcomes (González and Goldberg 2019).

Recently, gene regulation by non-coding RNAs, particularly long non-coding RNAs (LncRNAs), has emerged as a promising avenue for developing novel therapies for HS. LncRNAs regulate gene expression through multiple mechanisms, including epigenetic modulation, transcriptional interference, and mRNA degradation (Tu et al. 2018, Yan and Bu 2021). Taurine upregulated gene 1 (TUG1), a LncRNA located on chromosome 22, has been implicated in various cellular processes such as proliferation and apoptosis (Xiao et al. 2021). Previous studies have demonstrated the involvement of TUG1 in fibrotic diseases affecting the liver and heart (Zhu et al. 2021, Sun et al. 2021). In HS, TGU1 is upregulated, leading to fibrosis via the TAK1/YAP/TAZ pathway (Li et al. 2018, Wu et al. 2020). However, the upstream regulatory inputs controlling TUG1, as well as its downstream competing endogenous RNA (ceRNA) interactions in cutaneous fibrosis, remain unclear.

MicroRNAs (miRNAs) also play critical roles in HS. For instance, downregulation of miR-29 has been linked to scar formation, and therapeutic delivery of miR-29 mimics has shown potential to reduce fibrosis in HS models (Li et al. 2021). Other miRNAs, including miR-3613-3p, miR-627, miR-519d, and miR-6836-3p, have also been implicated in the regulation of fibroblast activity and extracellular matrix production (Li et al. 2021, Li et al. 2021, Yu et al. 2021, Zhou et al. 2018, Liu et al. 2018). Computational analysis using the StarBase database has predicted a direct interaction between TUG1 and miR-627. However, the existence of this interaction in HSFs and its functional consequences have not been experimentally confirmed.

Insulin-like Growth Factor Receptor 1 (IGF1R), a transmembrane tyrosine kinase receptor, has recently been identified as a key promoter of fibrosis and scar formation (Kim et al. 2012, Wang et al. 2020). Several miRNAs have been shown to modulate IGF1R signaling, and reciprocal regulatory feedback has been reported in fibrotic tissues (Xia et al. 2021, Guo et al. 2019). Notably, miR-627 is predicted to target IGF1R (Guo et al. 2019), although this interaction has not been studied in the context of HS. Whether TUG1 regulates IGF1R expression by sponging miR-627 remains unknown. The proposed TUG1/miR-627/IGF1R axis represents a novel regulatory pathway that has yet to be characterized in hypertrophic scar pathogenesis.

The present study aims to investigate the aberrant expression patterns of TUG1, miR-627, and their downstream target IGF1R in HS. Specifically, the expression patterns of these molecules in HS tissues, as well as their roles in modulating fibroblast proliferation and migration in vitro and in vivo will be examined. Based on bioinformatic predictions and prior findings in fibrosis, we hypothesize that LncRNA TUG1 promotes HS pathogenesis by acting as a molecular sponge for miR-627, thereby relieving miR-627–mediated suppression of IGF1R. Elucidating this novel TUG1/miR-627/IGF1R regulatory axis may provide new insights into the molecular pathogenesis of HS and identify potential targets for therapeutic intervention.

## Materials & methods

### Study subjects and sample collection

#### Inclusion and exclusion criteria

Inclusion criteria: (1) Patients without hematological disorders or systemic diseases. (2) Patients diagnosed with hypertrophic scar based on clinical presentation and response to prior treatment, in accordance with the diagnostic principles outlined in plastic surgery guidelines. Specifically, the scar must be elevated above the surrounding skin but confined within the original wound boundaries, thereby distinguishing it from keloid formation. (3) Patients who have not recieved other treatment methods for hypertrophic scarring prior to enrollment.

Exclusion criteria: Pregnant or lactating women.

#### Patient enrollment and tissue sample collection

This study enrolled 30 patients (19 males and 11 females, mean age 32.57 ± 1.32 years) who were diagnosed with hypertrophic scar (HS) and scheduled to undergo scar resection surgery in the Department of Plastic Surgery at Northern Theater General Hospital between May and October 2021. After obtaining written informed consent, HS tissues were collected during the surgery. Unaffected normal skin located at least 5 cm from the edge of the HS was also obtained from the resection margin of the same patients to serve as controls. An additional 30 patients (13 males and 17 females, mean age 33.15 ± 2.16 years) who underwent scar revision surgery donated normotrophic scar tissues located on the trunk, neck, face, and extremities. The study protocol was approved by the Ethics Committee of Northern Theater General Hospital.

#### LncRNA microarray analysis

Total RNA was extracted from hypertrophic scar, normotrophic scar, and adjacent normal skin tissues using Trizol reagent (Invitrogen, USA) according to the manufacturer’s protocol. The quality and concentration of RNA were assessed using a NanoDrop spectrophotometer. The RNA samples were then sent to Liaoning Baihao Company (Liaoning, China) for LncRNA microarray analysis, with each sample group analyzed in triplicate. Differentially expressed LncRNAs were identified by filtering the microarray data with the thresholds of absolute fold change > 2.0 and a p-value < 0.05. This initial screening yielded a list of 36 differentially expressed LncRNAs, which are visually represented in the heatmap. Subsequently, from this candidate list, the LncRNA TUG1 was selected for further investigation based on the following criteria: (1) it exhibited one of the most pronounced expression differences (both in terms of fold change and statistical significance) between hypertrophic scar and normal skin tissues; (2) it has been previously reported in the literature to be involved in processes closely related to tissue fibrosis, such as extracellular matrix deposition and fibroblast activation.

### Cell isolation, culture, and transfection

#### Isolation and primary culture of fibroblasts

Primary hypertrophic scar fibroblasts (HSFs) and normal skin fibroblasts (NSFs) were isolated from HS tissues and paired adjacent normal skin tissues, respectively. All tissues were collected under sterile conditions and thoroughly rinsed with phosphate-buffered saline (PBS) to remove residual blood and debris. Sample tissues were minced into approximately 1 mm³ fragments using sterile surgical blades, then incubated with 0.25% trypsin-EDTA (Gibco) at 37 °C for 30 min. Enzymatic digestion was neutralized by adding an equal volume of complete culture medium consisting of Dulbecco’s Modified Eagle Medium (DMEM; Gibco) supplemented with 10% fetal bovine serum (FBS; Gibco). The cell suspension was gently pipetted to facilitate single-cell dispersion, followed by centrifugation at 300×g for 5 min. After discarding the supernatant, the pellet was resuspended in fresh culture medium and passed through a sterile 100 μm nylon cell strainer (pretreated with 70% ethanol and rinsed with PBS) to remove undigested tissue. The filtered suspension was centrifuged again, and the resulting pellet was seeded into culture flasks. Cells were maintained at 37 °C in a humidified incubator with 5% CO₂, and the medium was changed every 2–3 days. Fibroblasts at passages 3 to 8 were used for subsequent experiments once 80% confluence was reached. Cell morphology was assessed by phase-contrast microscopy. Fibroblasts were identified based on their characteristic spindle-shaped appearance.

#### Transfection of plasmids/siRNA/miRNA mimics

Upon reaching 80% confluence, fibroblasts between passages 3–8 were trypsinized and seeded in 6-well plates at a density of 1 × 10^5^ cells/well for transfection. The TUG1 overexpression plasmid (pcDNA3.1-TUG1) and corresponding empty vector control (pcDNA3.1) were obtained from Sangon Biotech (Shanghai, China). Transfections were performed using Lipofectamine™ 2000 Transfection Reagent (1.5 mL; Cat. No. 11668019; Thermo Fisher Scientific, USA) according to the manufacturer’s instructions. Small interfering RNAs (siRNAs) were synthesized by RiboBio (Guangzhou, China). The sequence of the TUG1-targeting siRNA (si-TUG1) was 5′-TGAATTTCAATCATTTGAGAT-3′ (Cat. No. siB1097143801-1-5), and the negative control siRNA sequence was 5′-GCTCCCTTCAATCCAA-3′ (Cat. No. siN0000002-1-5). Both siRNAs were transfected at a final concentration of 100 nM. The miR-627 mimic and corresponding negative control mimic were also obtained from RiboBio and transfected at a final concentration of 50 nM. All transfections were conducted using Lipofectamine™ 2000 under serum-free conditions, and the medium was replaced with complete culture medium 6 h post-transfection. Cells were harvested 48 h after transfection for subsequent analyses.

### Molecular expression detection

#### Quantitative real-time PCR (qRT-PCR)

Total RNA was extracted from cultured fibroblasts using Trizol reagent (Invitrogen) according to the manufacturer’s instructions. RNA concentration and purity were measured by a NanoDrop 2000 spectrophotometer (Thermo Scientific). For mRNA and lncRNA detection, 1 µg of total RNA was reverse transcribed into cDNA using the PrimeScript RT Reagent Kit (Takara). For miRNA detection, reverse transcription was performed using stem-loop specific reverse transcription primers. Briefly, total RNA was mixed with the stem-loop RT primer and incubated at 42 °C for 3 min, followed by rapid cooling at 4 °C for at least 2 min. Then, 2.5 µL of reverse transcription reaction mix was added to the tube. The reaction mixture was incubated at 42 °C for 15 min to allow reverse transcription, followed by inactivation at 95 °C for 3 min. The resulting cDNA was stored at 4 °C for subsequent qPCR.

QRT-PCR was performed on an Applied Biosystems 7500 Fast Real-Time PCR System using SYBR Premix Ex Taq II (Takara).The primers used for this study are listed in Table 1. The reaction conditions were 95 °C for 30 s, followed by 40 cycles of 95 °C for 5 s and 60 °C for 30 s. GAPDH was used as the internal control for TUG1, IGF1R,α-SMA, and TGF-β while U6 snRNA was used for miR-627 normalization. The relative expression levels were calculated by the 2^−ΔΔCt^ method.

**Table 1:** Primer sequences used for qRT-PCR

Gene	Primer direction	Sequence (5′→3′)
TUG1	Forward	TAGGAGTGGATGTGTTCTGTAGCA
	Reverse	TGGTCGTGGAATATGGTCAATGAG
miR-627	Forward	TGTCAGAACTAGAATTTGATTATTAACATA
	Reverse	CAGTTCTTTGTTACAAAATGTACGTG
IGF1R	Forward	GCCCAAGGCTCAGAAGG
	Reverse	TTTAACAGGTAACTCGTGC
α-SMA	Forward	CATCACCAACTGGGACGACAT
	Reverse	ACAGAGTATTTGCGCTCCGAA
TGF-β	Forward	GGCCAGATCCTGTCCAAGC
	Reverse	GTGGGTTTCCACCATTAGCAC
GAPDH	Forward	CGACCACTTTGTCAAGCTCA
	Reverse	ACTGAGTGTGGCAGGGACTC
U6	Forward	CTCGCTTCGGCAGCACATATACT
	Reverse	ACGCTTCACGAATTTGCGTGTC

#### Luciferase reporter assay

To evaluate the direct interaction between miR-627 and the 3′ untranslated regions (3′-UTRs) of TUG1 and IGF1R, luciferase reporter plasmids were constructed. DNA fragments containing either the wild-type (WT) or mutant (MUT) predicted miR-627 binding sites were synthesized and subcloned into the pGL3-promoter luciferase vector (GeneCopoeia, USA). For the mutant constructs, 3–5 nucleotides within the miR-627 seed binding region were altered to disrupt the predicted base pairing. HSFs were seeded in 24-well plates and co-transfected with 100 ng of luciferase reporter plasmid, 50 nM miR-627 mimic or negative control mimic (NC-mimic) using Lipofectamine 2000. After 48 h of incubation, luciferase activity was measured using the Dual-Luciferase Reporter Assay System (YunCui Bio, Wuxi, China) according to the manufacturer’s protocol. Firefly luciferase activity was normalized to Renilla luciferase activity within the same well. Each transfection was performed in triplicate, and the experiment was repeated independently three times.

#### Western blotting

The total proteins of fibroblasts from HS samples were extracted with RIPA lysate and split on ice. BCA protein determination kit (Nanjing Nuoweizan Biotechnology Co., Ltd.) was used for quantitative analysis. The protein sample was dissolved and separated with 10% SDS-PAGE gel, and then transferred to PVDF membrane (Sigma-Aldrich, USA). Soak in TBST containing 5% skim milk powder and seal for 2 h. The membrane and the primary antibody were incubated overnight at 4 ℃, and then incubated with polyperoxidase-conjugated anti-mouse IgG secondary antibody for 2 h. Protein bands were detected by ECL Western blotting kit (Cytiva, Shanghai). The main antibodies used in this study are IGF1R (1:1000, mouse monoclonal, Cat# sc-462; Santa Cruz Biotechnology), α-SMA (1:1000, mouse monoclonal, Cat# ab7817; Abcam), TGF-β (1:1000, rabbit monoclonal, Cat# 3711; Cell Signaling Technology) and GAPDH (1:1500, mouse monoclonal, Cat# sc-47724; Santa Cruz Biotechnology). GAPDH was used as the internal control.

### Cellular function assays

#### MTT assay

Fibroblasts were inoculated in 96-well plate with a density of 1 × 10^3^ per well and divided into four groups. At 0, 12, 24, 36, and 48 h post-transfection, 10 µL of MTT solution (5 mg/mL; Beijing Yita Biotechnology Co., Ltd., China) was added to each well under light-protected conditions. Cells were then incubated at 37 °C in a 5% CO₂ incubator for 4 h. solution was added at 0, 12, 24, 36 and 48 h after transfection, 10 µl/well. After incubation, the supernatant was carefully removed, and 200 µL of dimethyl sulfoxide (DMSO, 0.5%; Beijing Yita Biotechnology Co., Ltd., China) was added to each well to dissolve the formazan crystals. Plates were gently shaken for 15 min to ensure complete solubilization. Absorbance was measured at 490 nm using a microplate reader. Optical density (OD) values were recorded, and relative cell proliferation rates were calculated accordingly. Each group was tested in triplicate, and the experiment was independently repeated three times.

#### Transwell assay

Fibroblasts were harvested using 0.25% trypsin, washed three times with sterile PBS, and resuspended in serum-free DMEM containing 10% FBS to achieve a uniform cell suspension. The cell density was adjusted appropriately, and 200 µl of the suspension was inoculated into the upper chanmer of a Transwell insert (8.0 μm pore size; 24-well format; Corning, USA). The upper chamber contained serum-free medium, while 600 µL of DMEM supplemented with 10% FBS was added to the lower chamber as a chemoattractant. After incubation at 37 °C with 5% CO₂ for 24 h, non-migrating cells on the upper surface of the membrane were removed gently with a cotton swab. The membranes were then fixed with 4% paraformaldehyde at room temperature (25 °C) for 30 min and stained with 1% (w/v) crystal violet solution for 20 min. Excess stain was removed by rinsing five times with PBS. The stained membranes were observed under an inverted microscope (×200), and cells that had migrated to the lower surface were counted. Five random fields per membrane were selected for quantification. Each group was tested in triplicate, and the experiment was repeated independently three times.

### Animal models

#### Construction of hypertrophic scar model by rabbit ear

A HS model was established using adult New Zealand white rabbits (*n* = 16, 2.5–3.0 kg), which were randomly assigned to four groups (*n* = 4 per group): (1) Negative control (NC, scrambled agomir), (2) TUG1 agomir, (3) miR-627 agomir, (4) TUG1 + miR-627 agomir. Rabbits were housed under standard laboratory conditions with free access to food and water. All animal procedures were approved by the Institutional Animal Care and Use Committee (IACUC) of the General Hospital of Northern Theater Command (R20210316) and were performed in accordance with institutional guidelines.

After general anesthesia, six full-thickness circular wounds (diameter ≈ 1 cm) were created on the ventral side of each ear using a sterile biopsy punch, spaced approximately 1 cm apart in two parallel rows. The perichondrium was preserved to induce hypertrophic scarring, and wounds were left to heal by secondary intention. One week post-wounding, local treatments were administered directly into the scar tissue via intradermal multipoint injection. Each wound received 50 µL of Lipofectamine 2000 (Thermo Fisher Scientific, Cat. No. 11668019)–encapsulated RNA complex per injection site. The injection formulation for each group was as follows: TUG1 group: TUG1 agomir (5 nmol/50 µL, final concentration 100 µM); miR-627 group: miR-627 agomir (5 nmol/50 µL, 100 µM); TUG1 + miR-627 group: mixed agomirs, 5 nmol each per 50 µL (1:1 molar ratio); NC group: scrambled agomir (5 nmol/50 µL, same formulation). Injections were performed using a microsyringe into multiple points within the scar tissue to ensure uniform distribution of the reagent. Treatments were administered once, and wounds were monitored daily for general condition and signs of infection. After three weeks, scar formation, epithelialization, and morphological changes were observed and recorded. Scar tissues were then harvested for histological and molecular analyses.

#### Hematoxylin and eosin (H&E) staining

Scar tissues from rabbit ears were fixed in 4% paraformaldehyde (PFA) for 24 h at room temperature and subsequently placed in tissue cassettes. Samples were dehydrated through a graded ethanol series (75%, 95%, and 100%), cleared in xylene, and embedded in paraffin wax. Paraffin-embedded tissues were sectioned at a thickness of 4 μm using a microtome (Leica, Germany). Tissue sections were dewaxed in xylene, rehydrated through a descending ethanol series (100%, 95%, 75%), and rinsed in distilled water. Sections were then stained with Harris’s hematoxylin for 5 min, differentiated in 1% acid alcohol (1% HCl in 70% ethanol) for 5 s, and blued under running tap water for 5 min. Subsequently, sections were counterstained with 1% aqueous eosin solution for 1 min, dehydrated through ascending ethanol gradients, cleared in xylene, and mounted with neutral balsam.

Stained sections were examined under a bright-field light microscope (Leica, Germany), and images were captured using a digital camera connected to the microscope and processed with Leica Application Suite software.

#### Masson staining

Paraffin-embedded scar tissue sections were stained using the Masson trichromatic staining kit (Solarbio, Cat. No. G1340, China) to evaluate collagen deposition. After dewaxing in xylene and rehydration through a graded ethanol series, sections were stained according to the manufacturer’s protocol. Briefly, nuclei were stained with Weigert’s iron hematoxylin, cytoplasm with acid fuchsin, and collagen fibers with aniline blue following phosphomolybdic-phosphotungstic acid treatment. Sections were then dehydrated, cleared in xylene, and mounted with neutral balsam. Stained sections were observed under a bright-field light microscope (Leica, Germany), and images were digitally recorded for further analysis.

#### Immunohistochemistry (IHC) staining

Paraffin-embedded HS tissue sections were subjected to IHC to detect IGF1R expression. After dewaxing in xylene and rehydration through a graded ethanol series, antigen retrieval was performed using heat-induced epitope retrieval. Sections were immersed in pH 6.0 citrate buffer (10 mM sodium citrate, 0.05% Tween-20), placed in a heat-resistant container, and heated to boiling in a microwave oven at medium-high power. A gentle boil was maintained for 10–15 min, followed by natural cooling to room temperature. Slides were then rinsed three times in PBS (pH 7.4), 5 min each. Endogenous peroxidase activity was blocked using 3% H_2_O_2_ for 10 min at room temperature. Non-specific binding was blocked using the blocking reagent provided in the detection kit. Sections were then incubated overnight at 4 °C in a humidified chamber with mouse monoclonal anti-IGF1R antibody (sc-462, 1:1500 dilution; Santa Cruz Biotechnology, USA). PBS was used in place of the primary antibody in the negative control group.

On the following day, slides were incubated sequentially with Reagent 2 (primary antibody enhancer) and Reagent 3 (polyperoxidase-conjugated anti-mouse/rabbit IgG polymer). Detection was performed using 3,3’-diaminobenzidine (DAB) substrate, which produced a tan to brown precipitate at antigen-positive sites. Slides were counterstained with Harris hematoxylin for nuclear visualization and blued under running tap water. The staining procedure was carried out using the UltraVision Quanto Detection System (Thermo Fisher Scientific, Cat. No. TL125-QHD), following the manufacturer’s protocol. Stained sections were examined under a bright-field light microscope (Leica, Germany), and representative images were captured for analysis.

### Statistical analysis

All statistical analyses were performed using SPSS software version 16.0 (IBM Corp., Armonk, NY, USA). Data were presented as the mean ± standard deviation (SD) from at least three independent experiments. Comparisons between two groups were conducted using the Student’s t-test, while multiple group comparisons were analyzed using one-way analysis of variance (ANOVA) or Fisher’s least significant difference (LSD) post hoc test, as appropriate. A P value less than 0.05 was considered statistically significant.

## Result

### LncRNA TUG1 is upregulated in HS and promotes a pro-fibrotic phenotype in HSFs

**Fig. 1:**
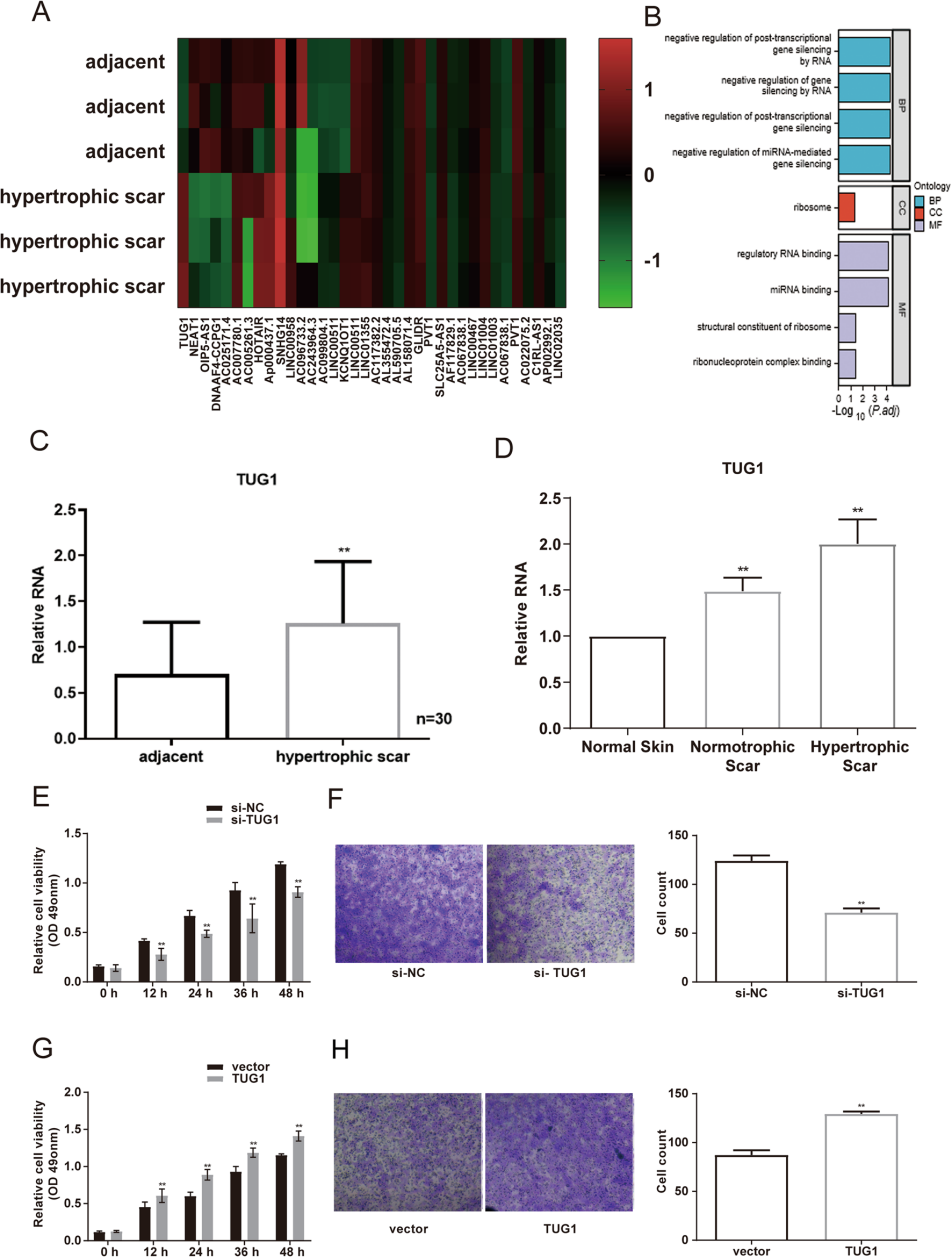
Upregulation of TUG1 in HS Promotes HSF Proliferation and Migration. **A** Heatmap representation from a Gene chip microarray analysis of differentially expressed lncRNAs in HS tissue compared to adjacent normal tissue. Red indicates upregulation; green indicates downregulation. **B** Gene Ontology (GO) enrichment analysis of the differentially expressed lncRNAs, showing representative terms for Biological Process (BP, blue), Cellular Component (CC, red), and Molecular Function (MF, purple). **C** TUG1 expression levels in adjacent normal tissue and HS tissue (*n* = 30), measured by qRT-PCR. **D** Bar graph comparing TUG1 expression across normal skin, normotrophic scar, and hypertrophic scar tissues. Data are presented as mean ± SD. *P* < 0.05 versus control (normal or adjacent tissue). **E** MTT assay showing reduced proliferation of HSFs following TUG1 knockdown. **F** Transwell migration assay demonstrating decreased migration of HSFs upon TUG1 knockdown. **G** MTT assay showing increased proliferation of HSFs after TUG1 overexpression. **H** Transwell migration assay showing enhanced HSF migration following TUG1 overexpression. Data are presented as mean ± SD. For (C) and (D), *n* = 30. For (E-H), experiments were performed in triplicate (*n* = 3). *P < 0.05, **P < 0.01 versus the respective control group

To delineate the potential role of TUG1 in HS, the expression levels of TUG1 was first examined in HS tissue. Gene expression profiling revealed that TUG1 expression was markedly elevated in hypertrophic scar (HS) tissues compared with adjacent tissues (Fig. 1A). To gain insights into the potential functional mechanisms of the dysregulated lncRNAs, GO enrichment analysis was performed. The results revealed significant enrichment in key biological processes, including “negative regulation of miRNA-mediated gene silencing” and “negative regulation of post-transcriptional gene silencing”. At the molecular function level, terms such as “miRNA binding” and “regulatory RNA binding” were prominently enriched (Fig. 1B). This finding was confirmed by qRT-PCR analysis, which showed significantly higher TUG1 levels in HS tissues relative to adjacent normal tissues (Fig. 1C). Further comparison among adjacent normal skin, normotrophic scar tissue, and HS tissue demonstrated that TUG1 expression was highest in HS samples (Fig. 1D), indicating a specific association between TUG1 upregulation and hypertrophic scarring.

Functional analysis was subsequently conducted to determine the effect of TUG1 on HSFs. Knockdown of TUG1 using siRNA significantly reduced HSFs proliferation and migration, as shown by MTT and Transwell assays (Fig. 1E, F). In contrast, TUG1 overexpression enhanced both proliferation and migration of HSFs (Fig. 1G, H), indicating a pro-fibrotic role of TUG1 in fibroblast behavior.

### TUG1 acts as a molecular sponge for miR-627

**Fig. 2:**
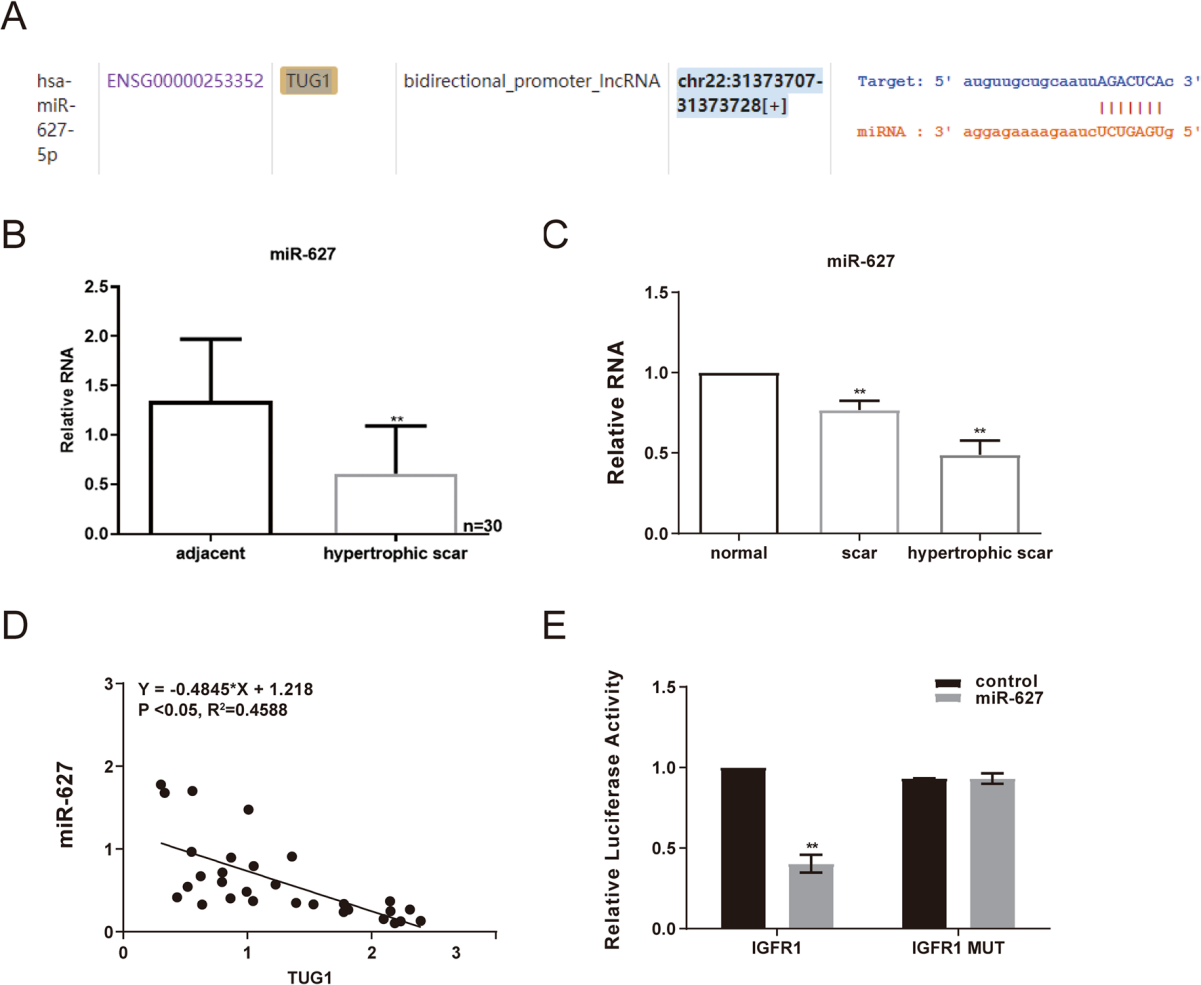
TUG1 negatively regulates miR-627 expression in HS. **A** Predicted binding sites between TUG1 and miR-627 identified via TargetScan. **B** qRT-PCR showing reduced miR-627 expression in HS tissues compared to adjacent normal tissues. **C** miR-627 expression across normal skin, normotrophic scar, and hypertrophic scar tissues. **D** Scatter plot and regression analysis indicating a negative correlation between TUG1 and miR-627 expression in clinical samples. **E** Luciferase reporter assay validating the direct interaction between TUG1 and miR-627 in HSFs. Reporter plasmids containing wild-type (WT) or mutant (MUT) TUG1 sequences were co-transfected with a miR-627 mimic or a negative control (NC) mimic. Data are shown as mean ± SD. **P < 0.01

TargetScan analysis predicted that TUG1 harbors a complementary binding site for miR-627 within its sequence (Fig. 2A). To examine the relationship between these two molecules, expression levels were assessed in hypertrophic scar (HS) tissues, adjacent normal tissues, and normotrophic scar tissues. Quantitative real-time PCR revealed that miR-627 expression was significantly downregulated in HS tissues compared to both adjacent normal tissues and normotrophic scar tissues (Fig. 2B, C). Pearson correlation analysis demonstrated a significant inverse correlation between TUG1 and miR-627 expression levels in 30 pairs of HS and adjacent tissue samples (Fig. 2D).

To determine whether TUG1 directly interacts with miR-627, a luciferase reporter assay was performed. The wild-type TUG1 sequence containing the predicted miR-627 binding site was cloned into a reporter vector, along with a mutant construct in which the seed region was altered. Co-transfection of HSFswith miR-627 mimic significantly suppressed luciferase activity in the wild-type group, whereas this suppressive effect was abolished in the mutant construct (Fig. 2E). These results indicate that TUG1 binds directly to miR-627 through a specific site and negatively regulates its activity.

### miR-627 directly targets and suppresses the pro-fibrotic gene IGF1R

**Fig. 3:**
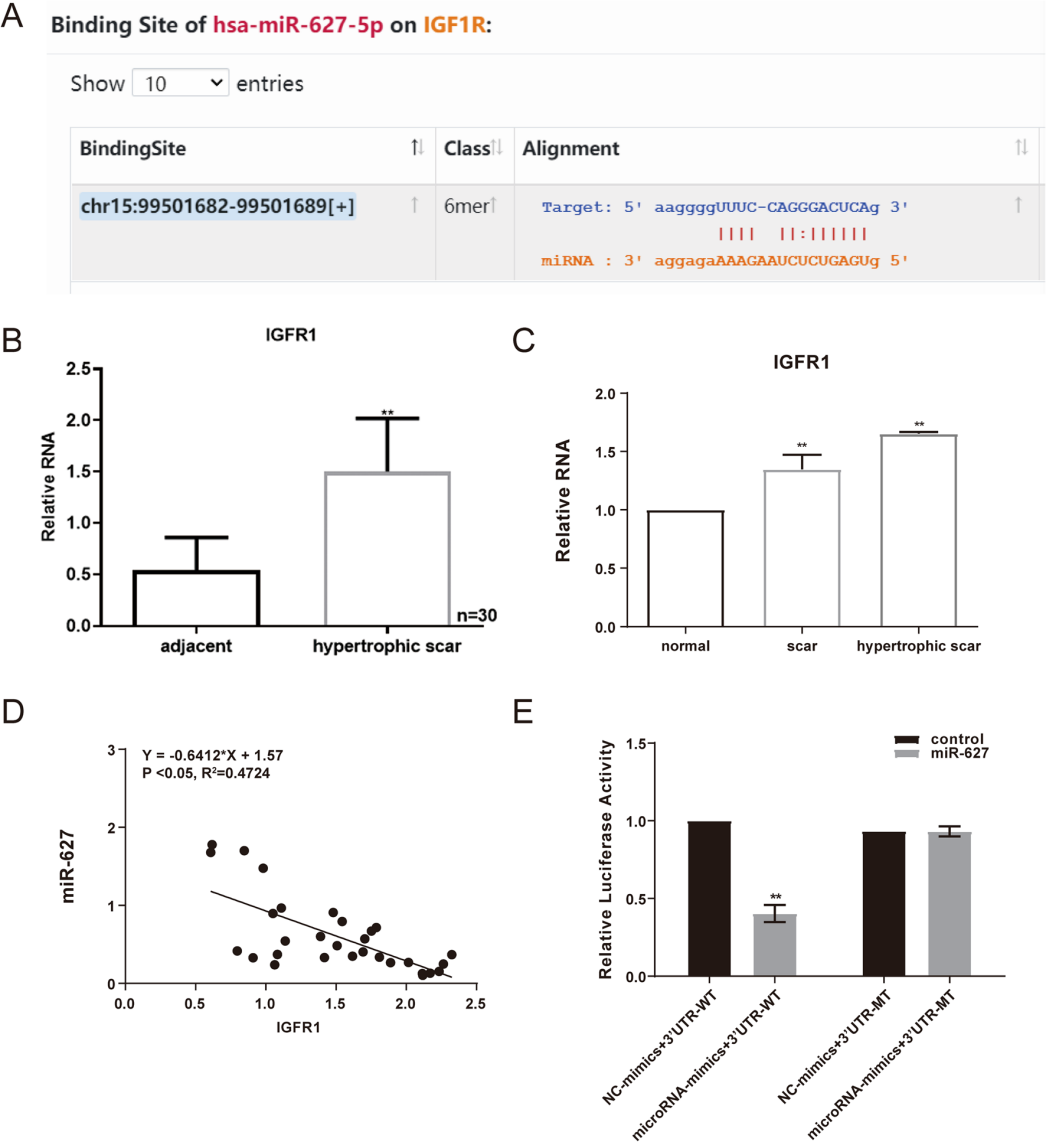
miR-627 directly targets and negatively regulates IGF1R. **A** Predicted binding sites between miR-627 and the 3′UTR of IGF1R identified using TargetScan. **B** qRT-PCR showing elevated IGF1R expression in HS tissues relative to adjacent normal tissues. **C** IGF1R mRNA levels in normal skin, normotrophic scar, and hypertrophic scar tissues. **D** Pearson correlation analysis revealing a negative correlation between miR-627 and IGF1R expression. **E** Luciferase reporter assay confirming that miR-627 directly binds to IGF1R 3′UTR; mutation of the binding site abolished the inhibitory effect. Data are presented as mean ± SD (*n* = 30). **P < 0.05 versus control (IGF1R + NC mimic)

In silico prediction revealed a putative miR-627 binding site within the 3′ untranslated region (3′-UTR) of the IGF1R transcript (Fig. 3A). qRT-PCR analysis showed that IGF1R expression was significantly upregulated in HS tissues compared to normal skin and normotrophic scar tissues (Fig. 3B, C). A significant inverse correlation was observed between miR-627 and IGF1R expression in clinical samples (Fig. 3D).

Subsequently, a luciferase reporter assay was performed using constructs containing either the wild-type or mutant IGF1R 3′-UTR to validate the direct interaction between miR-627 and IGF1R. Co-transfection of miR-627 mimic significantly reduced luciferase activity in the wild-type group. However, when the miR-627 binding site was mutated, this inhibitory effect was abolished (Fig. 3E). These findings confirm that miR-627 directly binds to the 3′-UTR of IGF1R and negatively regulates its expression.

### TUG1 exerts its pro-fibrotic function by regulating the miR-627/IGF1R axis

**Fig. 4:**
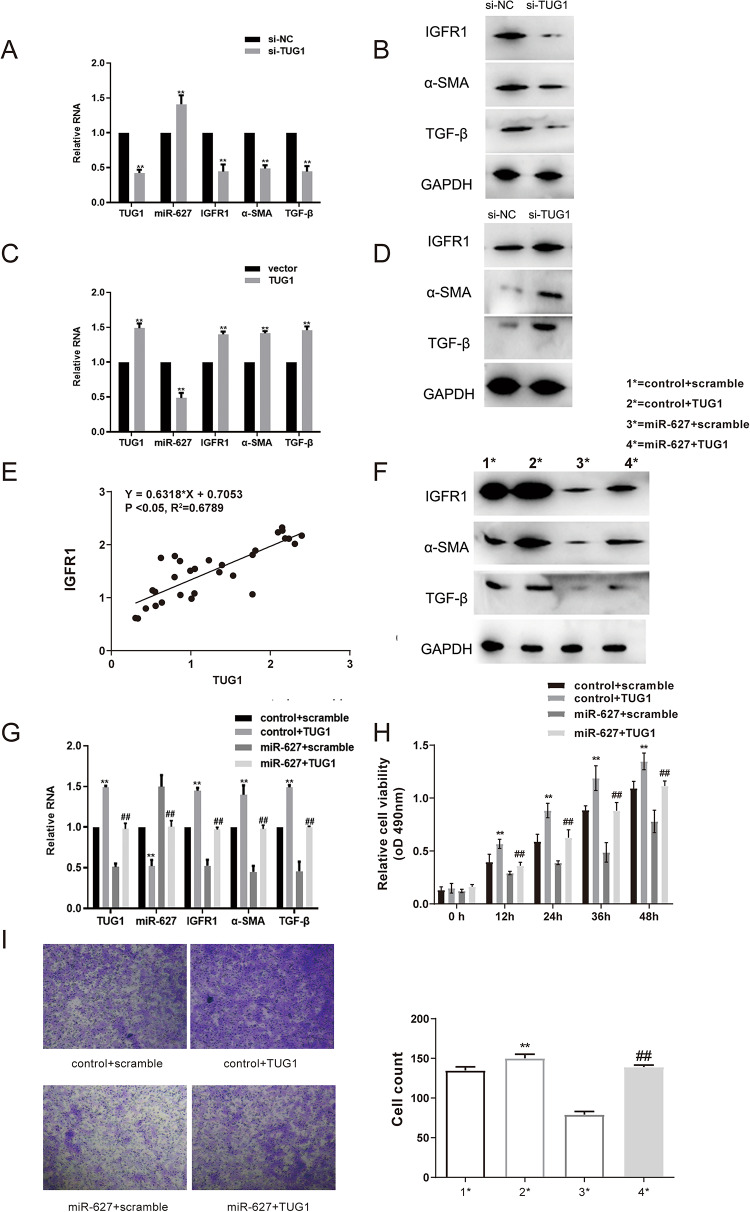
TUG1 promotes HSF proliferation and migration via the miR-627/IGF1R axis. **A** qRT-PCR analysis of TUG1, miR-627, IGF1R, α-SMA, and TGF-β expression in HSFs after transfection with si-TUG1 or si-NC. **B** Western blot analysis of IGF1R, α-SMA, and TGF-β protein levels after TUG1 knockdown. **C** qRT-PCR analysis of TUG1, miR-627, IGF1R, α-SMA, and TGF-β expression in HSFs after transfection with a TUG1 overexpression vector or an empty vector control. **D** Western blot analysis of IGF1R, α-SMA, and TGF-β protein levels after TUG1 overexpression. **E** Pearson correlation analysis showing a positive correlation between TUG1 and IGF1R mRNA levels in HS tissues (*n* = 30). **F** Western blot analysis of IGF1R, α-SMA, and TGF-β protein in rescue experiments. HSFs were co-transfected with combinations of control/TUG1 plasmids and scramble/miR-627 mimic as indicated. **G** qRT-PCR analysis of TUG1, miR-627, IGF1R, α-SMA, and TGF-β expression in the rescue experiments. **H** Cell proliferation assessed by MTT assay showing that miR-627 mimic reverses TUG1-induced HSF proliferation in the rescue experiment groups. **I** Cell migration assessed by Transwell assay showing that miR-627 mimic reverses TUG1-induced HSF migration in the rescue experiment groups. For in vitro experiments, data are presented as mean ± SD (*n* = 3). **P < 0.01 versus control+scramble group; ##*P* < 0.01 versus control+TUG1 group

To elucidate the molecular mechanism by which TUG1 promotes the pro-fibrotic phenotype, we first investigated its impact on the expression of its predicted targets, miR-627 and IGF1R, in HSFs. Following siRNA-mediated silencing of TUG1, qRT-PCR analysis revealed a significant increase in miR-627 expression and a concomitant decrease in IGF1R mRNA levels, along with reduced mRNA levels of the fibrosis markers α-SMA and TGF-β (Fig. 4A). This result at the transcript level was confirmed at the protein level, where Western blotting showed a marked reduction in IGF1R protein as well as α-SMA and TGF-β following TUG1 knockdown (Fig. 4B).

Conversely, in a complementary gain-of-function experiment, we overexpressed TUG1 in HSFs. This led to the opposite effects: TUG1 overexpression resulted in a significant suppression of miR-627 expression alongside a robust increase in IGF1R mRNA and the upregulation of α-SMA and TGF-β mRNA (Fig. 4C). Consistently, Western blot analysis demonstrated a substantial elevation of IGF1R, α-SMA, and TGF-β protein levels in TUG1-overexpressing cells (Fig. 4D). To determine if the regulatory link between TUG1 and IGF1R observed in vitro holds true in a clinical setting, we analyzed their expression in patient-derived hypertrophic scar tissues. A Pearson correlation analysis confirmed a significant positive correlation between TUG1 and IGF1R mRNA levels (Fig. 4E), supporting our in vitro findings.

Having established that TUG1 regulates IGF1R, we next performed rescue experiments to confirm that this regulation occurs specifically through the sponging of miR-627. As shown by Western blot, while TUG1 overexpression alone increased IGF1R, α-SMA, and TGF-β protein, this effect was substantially reversed when cells were co-transfected with a miR-627 mimic (Fig. 4F). The underlying transcriptomic changes supported this observation; qRT-PCR analysis showed that the miR-627 mimic effectively counteracted the TUG1-induced upregulation of IGF1R, α-SMA, and TGF-β mRNA (Fig. 4G).

Finally, we investigated whether this molecular axis was directly responsible for the observed cellular functions. TUG1’s ability to promote HSF proliferation was significantly blunted by the co-transfection of the miR-627 mimic, as measured by the MTT assay (Fig. 4H). Similarly, the Transwell migration assay revealed that the miR-627 mimic abrogated the pro-migratory effect induced by TUG1 overexpression (Fig. 4I).Taken together, these experiments demonstrate that TUG1 modulates IGF1R expression, drives myofibroblast activation (α-SMA), activates core fibrogenic signaling (TGF-β), and subsequent HSF proliferation and migration by acting as a molecular sponge for miR-627.

### TUG1 promotes hypertrophic scar formation in vivo

**Fig. 5:**
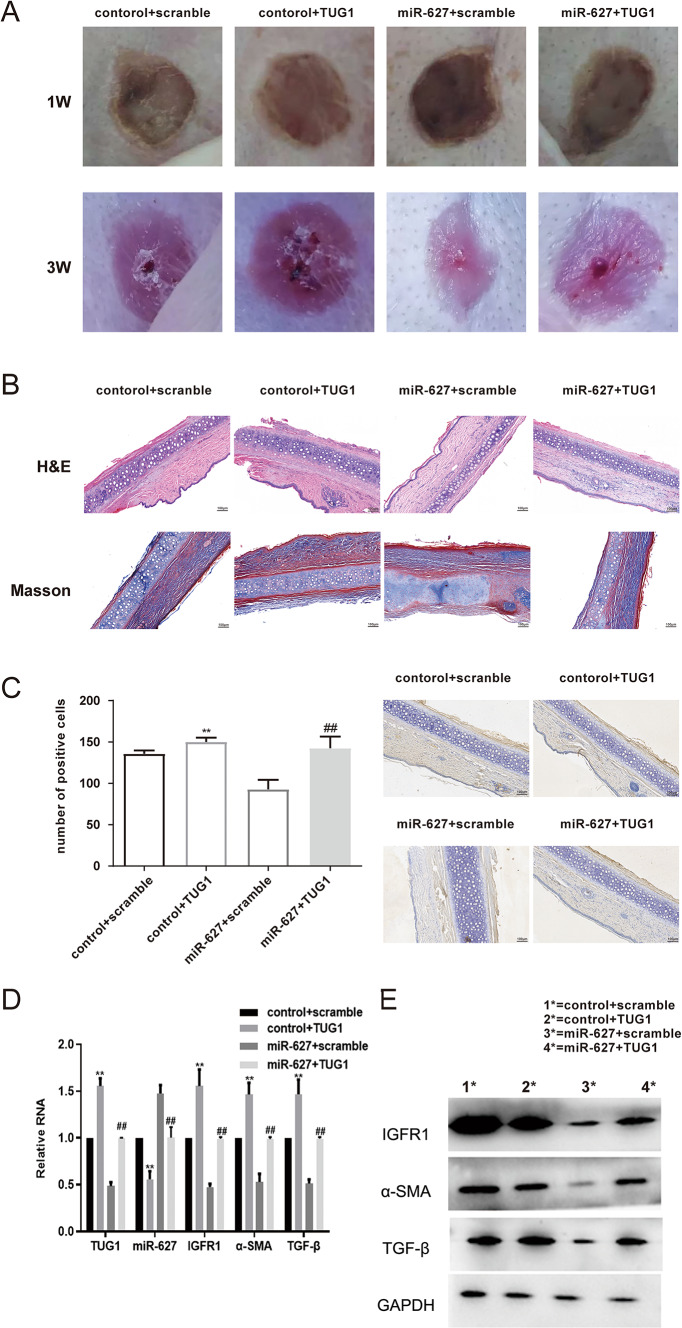
TUG1 promotes hypertrophic scar formation in vivo by suppressing miR-627. **A** Gross morphology of HS tissue at 1 and 3 weeks post-wounding under four transfection conditions. TUG1 overexpression enhanced HS size and thickness; miR-627 upregulation attenuated these effects. **B** Representative H&E and Masson staining images at 3 weeks showing increased dermal thickening and collagen deposition in TUG1 group, reduced in miR-627 group. **C** Immunohistochemical staining of IGF1R at 3 weeks. The TUG1 group shows increased staining, an effect that was reversed by co-treatment with miR-627 mimic. Scale bar = 50 μm. **D** Western blot showing elevated IGF1R, α-SMA, and TGF-β protein in TUG1 group and reduced levels in the miR-627 group. **E** qRT-PCR of scar tissue confirming that TUG1 upregulates IGF1R, α-SMA, and TGF-β and suppresses miR-627, while miR-627 overexpression reduces both TUG1 and IGF1R. Co-transfection restores levels close to baseline. Data are presented as mean ± SD (*n* = 30). **P < 0.05 versus control+scramble; ^^*P* < 0.05 versus control+TUG1; ##*P* < 0.05 versus miR-627 + scramble

Building upon the cellular findings, an in vivo rabbit ear model was employed to validate the regulatory role of the TUG1/miR-627/IGF1R axis in hypertrophic scar formation. After three weeks of modeling, gross morphological observations revealed distinct differences among the treatment groups. TUG1 overexpression led to prominent scar elevation, deeper erythema, and increased surface thickening compared to the control group, suggesting enhanced scar proliferation. In contrast, miR-627 overexpression resulted in flatter, paler, and less elevated scars, resembling normal skin. Notably, co-administration of TUG1 and miR-627 mitigated these effects, producing macroscopic features comparable to the negative control group (Fig. 5A).

Histological analysis using H&E and Masson’s trichrome staining further supported these findings. TUG1 overexpression caused marked epidermal thickening, increased collagen fiber deposition, and disorganized dermal architecture. Conversely, miR-627 overexpression was associated with reduced dermal thickening and more orderly collagen arrangement. In the co-transfection group, histological features were partially rescued, with scar thickness and collagen organization approaching control levels (Fig. 5B).

Immunohistochemical staining of IGF1R revealed differential expression across treatment groups. TUG1-overexpressing tissues displayed abundant IGF1R-positive staining, particularly in dermal fibroblasts, whereas miR-627 overexpression markedly reduced IGF1R expression. Importantly, co-transfection of TUG1 and miR-627 restored IGF1R staining to baseline levels, indicating an attenuated effect (Fig. 5C).

At the molecular level, qRT-PCR and Western blotting confirmed these trends. TUG1 overexpression increased IGF1R, α-SMA, and TGF-β mRNA and protein levels while suppressing miR-627 expression. miR-627 mimic treatment reversed these changes, downregulating IGF1R. Co-treatment restored miR-627 and IGF1R expression to levels similar to the control group (Figs. 5D–E).

In summary, these in vivo results demonstrate that TUG1 promotes the formation of HS by sponging miR-627, thereby derepressing IGF1R expression and driving excessive collagen deposition and tissue thickening.

## Discussion

This study identifies a novel regulatory axis involving lncRNA TUG1, miR-627, and IGF1R that drives the development of HS. TUG1 was found to be significantly upregulated in HS tissues and fibroblasts, where it promotes fibroblast proliferation and migration. Mechanistically, TUG1 functions as a competing endogenous RNA (ceRNA) by sponging miR-627, thereby relieving the miRNA-mediated repression of IGF1R, a known pro-fibrotic effector. In vitro rescue experiments and in vivo validation in a rabbit ear HS model further confirmed the functional relevance of this TUG1/miR-627/IGF1R axis in scar formation.

While prior work linked TUG1 to fibrosis in other systems like the liver and heart (Li et al. 2018, Sun et al. 2021), its role in dermal scarring was unknown. Our finding that TUG1 expression is increased in hypertrophic scar tissues and fibroblasts compared to normal skin parallels observations in other fibrotic diseases where LncRNAs are aberrantly dysregulated (Hadjicharalambous and Lindsay 2020). Using loss- and gain-of-function experiments, we demonstrate TUG1 enhances fibroblast proliferation, migration, and collagen production – eliciting quintessential pro-fibrotic effects. These results provide the first evidence that TUG1 overexpression promotes the activated fibroblast phenotype that drives hypertrophic scar pathogenesis.

Mechanistically, our findings establish miR-627 as a direct downstream target of TUG1, functioning as a key intermediary in the regulation of fibroblast activity. miRNAs represent critical regulators of fibrosis, and restoring miRNA expression shows promise for anti-fibrotic therapy (Tadokoro et al. 2021). Although the role of miR-627 in HS has not been previously characterized, existing literature in renal and pulmonary fibrosis models suggests that it exerts anti-fibrotic effects by targeting genes involved in matrix production and fibroblast activation (Li et al. 2019, Zhu et al. 2022). In our study, miR-627 expression was significantly downregulated in HS tissues, and this reduction correlated inversely with TUG1 expression. Dual-luciferase reporter assays confirmed that TUG1 directly binds to miR-627, acting as a molecular sponge to limit its availability. Functionally, overexpression of miR-627 counteracted the pro-fibrotic effects induced by TUG1, reducing fibroblast proliferation and migration in vitro. These results strongly support a model in which TUG1 promotes fibrotic remodeling in HS by sequestering miR-627, thereby suppressing its function. This study, to our knowledge, is the first to identify miR-627 as a functional target of TUG1 and implicate it in dermal fibrogenesis.

Given that miRNAs typically function by repressing target mRNAs (Rupaimoole and Slack 2017), we next searched for downstream effectors of miR-627 using in silico prediction and identified IGF1R. The IGF1 pathway is implicated in fibrosis, including stimulating fibroblast collagen synthesis (Chung et al. 2021, Liu et al. 2021). We demonstrate miR-627 directly binds and suppresses IGF1R, with an inverse correlation between their expression in patient samples. Importantly, TUG1 induces IGF1R in a miR-627 dependent manner to elicit pro-fibrotic effects. This axis provides mechanistic insight into the pro-fibrotic function of TUG1 in hypertrophic scar formation and identifies potential therapeutic targets, such as miR-627 restoration or IGF1R inhibition, to counteract TUG1-mediated fibrosis.The pathological role of TUG1 is not confined to dermal fibrosis. It has been extensively documented as an oncogenic driver in various cancers, including hepatocellular carcinoma and non-small cell lung cancer, where it promotes proliferation, metastasis, and chemoresistance through diverse molecular sponges (Chung et al. 2021, Liu et al. 2021). In the context of fibrosis, beyond cardiac and hepatic systems, TUG1 upregulation has also been observed in pulmonary and renal fibrosis, often correlating with disease severity (Chung et al. 2021, Liu et al. 2021). Notably, the trachea, as a structure susceptible to fibrotic stenosis following injury or intubation, shares common fibroproliferative pathways with the skin. Although direct evidence in tracheal fibrosis is currently limited, the established role of TUG1 in pulmonary fibrosis and its regulation of key fibroblast behaviors suggest its potential involvement in tracheal scar formation, warranting future investigation.

Critically, we provide in vivo validation using a rabbit hypertrophic scar model. Local TUG1 delivery increased scar formation, collagen deposition, fibroblast proliferation, and IGF1R expression – effects partially rescued by co-delivering miR-627. To our knowledge, this represents the first demonstration of manipulating a ncRNA network to mitigate scarring in an animal model. These preclinical findings reveal the translational potential of silencing TUG1 or restoring miR-627 as an anti-fibrotic approach.

The broad implication of TUG1 across diseases underscores its potential as both a diagnostic biomarker and a therapeutic target. In HS, detecting TUG1 levels in tissue or serum could aid in scar assessment and prognosis prediction. Therapeutically, strategies could include: (1) directly targeting TUG1 with antisense oligonucleotides (ASOs) or small interfering RNAs (siRNAs); (2) administering miR-627 mimics to restore its anti-fibrotic function; or (3) employing IGF1R inhibitors to block the downstream effector pathway. The success of RNA-targeted therapies in other fields provides a promising precedent for such approaches in scar management.

Nonetheless, our study has certain limitations that provide avenues for future work. The exclusive use of male rabbits limits generalizability, and future studies should include both sexes. Temporal profiling of molecular changes throughout scar development could provide deeper insight into pathogenesis. While IGF1R was implicated as a downstream effector, direct inhibition studies are needed to confirm its functional role. Validation in additional models, such as porcine or human skin, would enhance translational relevance. Moreover, the upstream signals driving TUG1 expression remain unclear and merit exploration.

In summary, this study identifies lncRNA TUG1 as a key promoter of hypertrophic scarring through its regulation of the miR-627/IGF1R axis. TUG1 is upregulated in hypertrophic scar tissue and enhances fibroblast proliferation and migration by suppressing miR-627, leading to derepression of IGF1R. Functional rescue experiments in vitro and in vivo confirm this regulatory pathway’s role in driving fibrotic outcomes. These findings provide new insight into the molecular mechanisms of scar formation and suggest that targeting the TUG1–miR-627–IGF1R axis may offer a novel therapeutic strategy for HS treatment.

## Data Availability

No datasets were generated or analysed during the current study.

## References

[CR2] Barone N, Safran T, Vorstenbosch J, Davison PG, Cugno S, Murphy AM (2021) Current advances in hypertrophic scar and keloid management. Semin Plast Surg 35:145–152. 10.1055/s-0041-1731461 34526861 10.1055/s-0041-1731461 PMC8432993

[CR29] Chung EJ, Kwon S, Reedy JL, White AO, Song JS, Hwang I, Chung JY, Ylaya K, Hewitt SM, Citrin DE (2021) IGF-1 receptor signaling regulates type II pneumocyte senescence and resulting macrophage polarization in lung fibrosis. Int J Radiat Oncol Biol Phys 110:526–538. 10.1016/j.ijrobp.2020.12.035 33385497 10.1016/j.ijrobp.2020.12.035 PMC8784947

[CR3] Finnerty CC, Jeschke MG, Branski LK, Barret JP, Dziewulski P, Herndon DN (2016) Hypertrophic scarring: the greatest unmet challenge after burn injury. Lancet 388:1427–1436. 10.1016/s0140-6736(16)31406-4 27707499 10.1016/S0140-6736(16)31406-4 PMC5380137

[CR5] González N, Goldberg DJ (2019) Update on the treatment of scars. J Drugs Dermatol 18:550–555 31251547

[CR22] Guo L, Bai Y, Ji S, Ma H (2019) MicroRNA–98 suppresses cell growth and invasion of retinoblastoma via targeting the IGF1R/k–Ras/Raf/MEK/ERK signaling pathway. Int J Oncol 54:807–820. 10.3892/ijo.2019.4689 30664191 10.3892/ijo.2019.4689 PMC6365030

[CR24] Hadjicharalambous MR, Lindsay MA (2020) Idiopathic Pulmonary fibrosis: pathogenesis and the emerging role of long non-coding RNAs. Int J Mol Sci 21:524. 10.3390/ijms21020524 31947693 10.3390/ijms21020524 PMC7013390

[CR19] Kim YH, Sumiyoshi S, Hashimoto S, Masago K, Togashi Y, Sakamori Y, Okuda C, Mio T, Mishima M (2012) Expressions of insulin-like growth factor receptor-1 and insulin-like growth factor binding protein 3 in advanced non-small-cell lung cancer. Clin Lung Cancer 13:385–390. 10.1016/j.cllc.2011.11.009 22285568 10.1016/j.cllc.2011.11.009

[CR26] Li J, Kong X, Jiang S, Liao W, Zhang Z, Song J, Liang Y, Zhang W (2019) miR-627/HMGB1/NF-κB regulatory loop modulates TGF-β1-induced pulmonary fibrosis. J Cell Biochem 120:2983–2993. 10.1002/jcb.27038 30536600 10.1002/jcb.27038

[CR15] Li L, Han W, Chen Y, Chen Y (2021) MiR-3613-3p inhibits hypertrophic scar formation by down-regulating arginine and glutamate-rich 1. Mol Cell Biochem 476:1025–1036. 10.1007/s11010-020-03968-4 33165823 10.1007/s11010-020-03968-4

[CR11] Li T, Chen Y, Zhang J, Liu S (2018) LncRNA TUG1 promotes cells proliferation and inhibits cells apoptosis through regulating AURKA in epithelial ovarian cancer cells. Med (Baltim) 97:e12131. 10.1097/md.0000000000012131 10.1097/MD.0000000000012131 PMC6133603 30200102

[CR30] Liu B, Lin L, Yu S, Xia R, Zheng L (2021) Long non-coding RNA H19 acts as a microRNA-194 sponge to inhibit the apoptosis and promote the proliferation of hypertrophic scar fibroblasts. Can J Physiol Pharmacol 99:1288–1297. 10.1139/cjpp-2021-0351 34310900 10.1139/cjpp-2021-0351

[CR18] Liu F, Chen WW, Li Y, Zhang JQ, Zheng QB (2018) MiR-6836-3p promotes proliferation of hypertrophic scar fibroblasts by targeting CTGF. Eur Rev Med Pharmacol Sci 22:4069–4074. 10.26355/eurrev_201807_15396 30024593 10.26355/eurrev_201807_15396

[CR13] Li XM, Yu WY, Chen Q, Zhuang HR, Gao SY, Zhao TL (2021) LncRNA TUG1 exhibits pro-fibrosis activity in hypertrophic scar through TAK1/YAP/TAZ pathway via miR-27b-3p. Mol Cell Biochem 476:3009–3020. 10.1007/s11010-021-04142-0 33791919 10.1007/s11010-021-04142-0

[CR14] Li Y, Zhang J, Shi J, Liu K, Wang X, Jia Y, He T, Shen K, Wang Y, Liu J et al (2021) Exosomes derived from human adipose mesenchymal stem cells attenuate hypertrophic scar fibrosis by miR-192-5p/IL-17RA/Smad axis. Stem Cell Res Ther 12:221. 10.1186/s13287-021-02290-0 33789737 10.1186/s13287-021-02290-0 PMC8010995

[CR4] Nischwitz SP, Rauch K, Luze H, Hofmann E, Draschl A, Kotzbeck P, Kamolz L-P (2020) Evidence-based therapy in hypertrophic scars: an update of a systematic review. Wound Repair Regen 28:656–665. 10.1111/wrr.12839 32506727 10.1111/wrr.12839 PMC7539946

[CR1] Oosterhoff TCH, Beekman VK, van der List JP, Niessen FB (2021) Laser treatment of specific scar characteristics in hypertrophic scars and keloid: a systematic review. J Plast Reconstr Aesthet Surg 74:48–64. 10.1016/j.bjps.2020.08.108 33645505 10.1016/j.bjps.2020.08.108

[CR28] Rupaimoole R, Slack FJ (2017) MicroRNA therapeutics: towards a new era for the management of cancer and other diseases. Nat Rev Drug Discov 16:203–222. 10.1038/nrd.2016.246 28209991 10.1038/nrd.2016.246

[CR10] Sun Q, Luo M, Gao Z, Han X, Yan Z, Xie S, Zhao H, Sun H (2021) TUG1 knockdown suppresses cardiac fibrosis after myocardial infarction. Mamm Genome 32:435–442 34341870 10.1007/s00335-021-09895-z

[CR23] Sun Q, Luo M, Gao Z, Han X, Yan Z, Xie S, Zhao H, Sun H (2021) TUG1 knockdown suppresses cardiac fibrosis after myocardial infarction. Mamm Genome 32:435–442. 10.1007/s00335-021-09895-z 34341870 10.1007/s00335-021-09895-z

[CR25] Tadokoro T, Morishita A, Masaki T (2021) Diagnosis and therapeutic management of liver fibrosis by MicroRNA. Int J Mol Sci 22:8139. 10.3390/ijms22158139 34360904 10.3390/ijms22158139 PMC8347497

[CR6] Tu L, Huang Q, Fu S, Liu D (2018) Aberrantly expressed long noncoding RNAs in hypertrophic scar fibroblasts in vitro: A microarray study. Int J Mol Med 41:1917–1930. 10.3892/ijmm.2018.3430 29393369 10.3892/ijmm.2018.3430 PMC5810216

[CR20] Wang X, Chen L, Zhao X, Xiao L, Yi S, Kong Y, Jiang Y, Zhang J (2020) A cathelicidin-related antimicrobial peptide suppresses cardiac hypertrophy induced by pressure overload by regulating IGFR1/PI3K/AKT and TLR9/AMPKα. Cell Death Dis 11:96. 10.1038/s41419-020-2296-4 32029708 10.1038/s41419-020-2296-4 PMC7005284

[CR12] Wu X, Zheng X, Cheng J, Zhang K, Ma C (2020) LncRNA TUG1 regulates proliferation and apoptosis by regulating miR-148b/IGF2 axis in ox-LDL-stimulated VSMC and HUVEC. Life Sci 243:117287. 10.1016/j.lfs.2020.117287 31926240 10.1016/j.lfs.2020.117287

[CR21] Xia J, Li S, Ma D (2021) MicroRNA293p regulates the betacatenin pathway by targeting IGF1 to inhibit the proliferation of prolactinoma cells. Mol Med Rep 23:436 33846792 10.3892/mmr.2021.12071 PMC8060803

[CR8] Xiao M, Zou X, Li B, Zhang B (2021) Long non-coding RNA H19 promotes the proliferation, migration and invasion while inhibits apoptosis of hypertrophic scarring fibroblasts by targeting miR-3187-3p/GAB1 axis. Burns 47:654–664. 10.1016/j.burns.2020.07.023 32888745 10.1016/j.burns.2020.07.023

[CR7] Yan H, Bu P (2021) Non-coding RNA in cancer. Essays Biochem 65:625–639. 10.1042/ebc20200032 33860799 10.1042/EBC20200032 PMC8564738

[CR16] Yu J, Zhang L, Zhang S, Xian G, Zhao Y, Bu X (2021) MiR-29b inhibits hypertrophic scar tissue inflammation after burn through regulating TGF-β1/Smad signaling pathway. Ital J Dermatol Venerol 156:251–252. 10.23736/s2784-8671.19.06444-7 31578834 10.23736/S2784-8671.19.06444-7

[CR17] Zhou X, Xie Y, Xiao H, Deng X, Wang Y, Jiang L, Liu C, Zhou R (2018) MicroRNA-519d inhibits proliferation and induces apoptosis of human hypertrophic scar fibroblasts through targeting Sirtuin 7. Biomed Pharmacother 100:184–190. 10.1016/j.biopha.2018.01.158 29428666 10.1016/j.biopha.2018.01.158

[CR9] Zhu H-D, Lyu C-K, Ma F-F (2021) Effects of TUG1 on hepatic fibrosis and its mechanism. Zhongguo ying yong sheng li xue za zhi= Zhongguo yingyong shenglixue zazhi=. Chin J Appl Physiol 37:616–621 10.12047/j.cjap.6139.2021.094 34821094

[CR27] Zhu Y, Zha F, Tang B, Ji TT, Li XY, Feng L, Bai SJ (2022) Exosomal hsa_circ_0125310 promotes cell proliferation and fibrosis in diabetic nephropathy via sponging miR-422a and targeting the IGF1R/p38 axis. J Cell Mol Med 26:151–162. 10.1111/jcmm.17065 34854210 10.1111/jcmm.17065 PMC8742240

